# Mechanisms of Oxidative Damage in Multiple Sclerosis and Neurodegenerative Diseases: Therapeutic Modulation via Fumaric Acid Esters

**DOI:** 10.3390/ijms130911783

**Published:** 2012-09-18

**Authors:** De-Hyung Lee, Ralf Gold, Ralf A. Linker

**Affiliations:** 1 Department of Neurology, University of Erlangen; Schwabachanlage 6, Erlangen 91054, Germany; E-Mail: ralf.linker@uk-erlangen.de; 2 Department of Neurology, Ruhr University Bochum, Gudrunstr. 56, Bochum 44791, Germany; E-Mail: ralf.gold@ruhr-uni-bochum.de

**Keywords:** Fumaric acid ester, Nrf2, neurodegeneration, oxidative stress, multiple sclerosis, cytoprotektive

## Abstract

Oxidative stress plays a crucial role in many neurodegenerative conditions such as Alzheimer’s disease, amyotrophic lateral sclerosis and Parkinson’s as well as Huntington’s disease. Inflammation and oxidative stress are also thought to promote tissue damage in multiple sclerosis (MS). Recent data point at an important role of anti-oxidative pathways for tissue protection in chronic-progressive MS, particularly involving the transcription factor nuclear factor (erythroid-derived 2)-related factor 2 (Nrf2). Thus, novel therapeutics enhancing cellular resistance to free radicals could prove useful for MS treatment. Here, fumaric acid esters (FAE) are a new, orally available treatment option which had already been tested in phase II/III MS trials demonstrating beneficial effects on relapse rates and magnetic resonance imaging markers. *In vitro*, application of dimethylfumarate (DMF) leads to stabilization of Nrf2, activation of Nrf2-dependent transcriptional activity and abundant synthesis of detoxifying proteins. Furthermore, application of FAE involves direct modification of the inhibitor of Nrf2, Kelch-like ECH-associated protein 1. On cellular levels, the application of FAE enhances neuronal survival and protects astrocytes against oxidative stress. Increased levels of Nrf2 are detected in the central nervous system of DMF treated mice suffering from experimental autoimmune encephalomyelitis (EAE), an animal model of MS. In EAE, DMF ameliorates the disease course and improves preservation of myelin, axons and neurons. Finally, Nrf2 is also up-regulated in the spinal cord of autopsy specimens from untreated patients with MS, probably as part of a naturally occurring anti-oxidative response. In summary, oxidative stress and anti-oxidative pathways are important players in MS pathophysiology and constitute a promising target for future MS therapies like FAE.

## 1. Introduction: Oxidative Stress in Neurologic Diseases

In almost every chronic neurologic disease, spanning from inflammatory backgrounds to underlying primary degenerative processes, oxidative stress may play a major functional role in pathogenesis of disease initiation and progression.

In general, oxidative stress is generated by the inability to detoxify or to repair the resulting damage caused by the generation of reactive oxygen species (ROS) like superoxide (O_2_^−^), hydrogen peroxide (H_2_O_2_) or hydroxyl radicals (·OH). A dysbalance in the physiological redox state of a cell may thus lead to toxic effects via production of peroxides or free radicals that damage sub-cellular structures including proteins, lipids, and DNA. Further, some reactive oxidative species have the capacity to act as intracellular messengers in redox signaling cascades thus interfering with normal cellular signaling pathways. Additionally, reactive nitrogen species (RNS) are a family of toxic molecules derived from nitric oxide (·NO) and superoxide (O_2_^−^) produced via the enzymatic activity of inducible NO synthase 2 (NOS2) and NADPH oxidase, respectively. In concert, these molecules may eventually lead to oxidation or nitrosylation finally resulting in cellular damage, associated with cell death and subsequent organ dysfunction.

In eukaryotic organisms, a major source of reactive oxygen species is the process of oxidative phosphorylation in mitochondria. Mitochondria are multi-functional organelles whose function is not only restricted to the production of ATP through the respiratory chain. In particular, several redox-active flavoproteins as well as xanthine oxidase, NADPH oxidases and cytochrome P450 all contribute to the production of oxidants like e.g., superoxide. Additionally, hydrogen peroxide is produced by a wide variety of enzymes including several cellular oxidases. In the central nervous system (CNS), the (mal) function of mitochondria might render subsets of selectively vulnerable neurons intrinsically susceptible to cellular stress, aging and eventually death, in some cases depending on underlying genetic variations.

Over the past two decades, the pivotal role of mitochondrial biology for adult onset neurodegenerative disorders has emerged in different diseases. Such disorders may comprise Alzheimer’s disease (AD), amyotrophic lateral sclerosis (ALS), Parkinson’s disease (PD) as well as Huntington’s disease (HD). Even in vitamin deficiency, evidence indicates that oxidative stress, glutamate excitotoxicity and inflammation are major contributors to the resulting neuropathology, as for example shown in thiamine deficiency. In this condition, increased production of ROS is observed in the brain [1]. In Wernicke encephalopathy, oxidative stress is related to the underlying CNS pathology as evidenced by increased neuronal peroxidase activity. Consistent with the role of oxidative stress in thiamine deficiency, further changes include an increased expression of heme oxygenase (HO)-1 and intercellular adhesion molecule (ICAM)-1 as well as microglia activation [2,3].

## 2. Role of Endogenous Anti-Oxidative Pathways: The Importance of the Transcription Factor Nuclear Factor (Erythroid-Derived 2)-Related Factor 2 (Nrf2)

**Figure 1:**
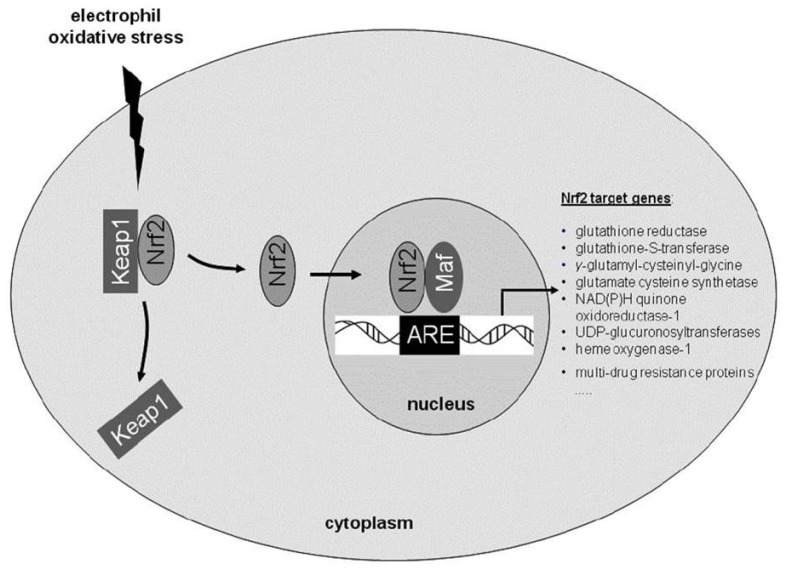
Scheme depicting the activation of the anti-oxidant transcription factor nuclear factor (erythroid-derived 2)-related factor 2 (Nrf2) including selected target genes presumably involved in anti-oxidant responses.

Over the past decades, several exogenous compounds have been suggested as anti-oxidative treatment approaches, many with ambiguous clinical success. In the recent years, the characterization of endogenous cellular anti-oxidative responses entered the focus of interest, e.g., signaling pathways involving nuclear factor (erythroid-derived 2)-related factor 2 (Nrf2). Nrf2 is a redox-sensitive basic leucine zipper transcription factor which possesses a domain for interaction with the cytoplasmatic protein kelch like ECH associated protein (Keap-1) [4]. Upon disruption of the keap1 gene, constitutive activation of Nrf2 and its targeted genes causes juvenile lethality due to hyperkeratotic lesions in the esophagus and rodent fore- stomach. *In vivo* evidence of a functional interaction between Nrf2 and Keap1 has been demonstrated in a setting where the lethality of keap1 deficiency is reversed by the parallel knockout of the nrf2 gene [5]. Under basal conditions, Nrf2 remains in the cytoplasm, associated with the actin cytoskeleton through Keap1 [4]. In the status of homeostasis, Nrf2 is rapidly degraded and displays a half-life between 10 and 40 min [6,7]. In the presence of oxidative stress or electrophilic compounds, the Nrf2-Keap1 interaction is abolished via disruption of distinct cysteine residues in Keap1. Subsequently, Nrf2 translocates into the nucleus, where it dimerizes with small Maf proteins (musculoaponeurotic fibrosarcoma oncogene homologues) to increase the transcription rate of antioxidative response element (ARE)-driven genes (Figure 1). Thus, Nrf2 activation can inhibit or diminish cellular damage in different tissues and organs [8]. Nrf2 associated protective effects depend on the coordinated expression of genes with detoxifying, anti-oxidant capabilities. Here, two enzymatic systems are particularly important for the prevention of oxidative damage in cells of the nervous system: the heme oxygenase system and a group of enzymes involved in glutathione synthesis and utilization.

First, HO is a microsomal enzyme with two isoforms: a constitutive isoform HO-2 and an inducible enzyme HO-1 [9,10]. In the CNS of rodents, HO-2 is nearly ubiquitously expressed. In contrast, in the normal brain, basal HO-1 expression is confined to small groups of scattered neurons and glial cells [11]. The induction of HO-1 is considered to counteract oxidative damage and confer cytoprotection, as suggested by studies with either deficiency in, or overexpression, of HO-1 [12–14]. In the nervous system, HO-1 can be activated in glial cells by its substrate heme, via Nrf2 activation, and by a plethora of pro-oxidant and inflammatory stimuli [15–17].

Second, superoxide dismutase (SOD) activity plays a major role in the process of radical detoxification by converting superoxide to hydrogen peroxide. The efficacy of the SOD system relies on the subsequent decomposition of hydrogen peroxide by catalase or glutathione peroxidase to inhibit the conversion of hydrogen peroxide to the hydroxyl radical. Here, the peroxisomal catalase quickly metabolizes peroxide into water and molecular oxygen. Yet, the specific activity of catalase in the brain is much lower than in peripheral tissues [18,19]. Thus, glutathione peroxidase has a major role in the disposition of hydrogen peroxide and organic hydroperoxides in CNS tissue. Here, astrocytes harbor a more efficient detoxification and anti-oxidative potential than neurons. On a cellular level, catalase is not expressed in mitochondria. In this organelle, glutathione is mainly responsible for detoxification during physiological or pathological conditions.

Well in line with a pivotal role of Nrf2 for ROS detoxification, Nrf2 deficient cells are more sensitive to peroxides, NO, mitochondrial toxins, endoplasmatic reticulum stress and glucose deprivation [8]. Although Nrf2 knock-out mice apparently develop normally, aged mice are prone to autoimmune diseases: Female Nrf2 deficient mice older than 60 weeks of age develop a lupus-like severe nephritis, characterized by cellular proliferation, lobar formation, and massive granular deposits of IgG, IgM and C3 along the capillary walls. In this context, Nrf2 and consequently the oxidative cellular response may constitute one of the factors which influences the susceptibility towards autoimmune diseases [20]. Moreover, Nrf2 is also a regulator of innate immune responses. Disruption of the nrf2 gene leads to dramatically increased mortality rates in response to pro-inflammatory challenges as mediated e.g., by LPS as well as TNF-α [21]. Consequently, Nrf2 deficient mice are more sensitive to pulmonary inflammatory diseases, chemical hepatotoxicity and carcinogenesis [22]. On a molecular level, Nrf2 dependent glutathione levels regulate the sensitivity of cells to Fas and TNF-α induced apoptosis [23,24]. There is an inverse correlation between glutathione content and the ability to initiate apoptotic downstream signaling pathways, suggesting that glutathione levels regulate the susceptibility of the cell towards death receptor signals. Accordingly, increased glutathione levels mediated by Nrf2 completely prevent p75 neurotrophin receptor and Fas mediated motor neuron apoptosis [25].

## 3. Oxidative Stress and the Nrf2 Pathways in Neurodegenerative Diseases

PD, ALS as well as AD and HD are common human neurodegenerative diseases. They are characterized by neurodegeneration in specific, susceptible anatomical CNS regions. While genetic forms of PD, ALS and AD provide important clues for the respective disease pathogenesis, the exact sequence of events leading to the more common sporadic forms of these disorders are still not clear to date. While a secondary involvement of the immune system may play an important role in disease pathogenesis, all conditions are mainly considered as primarily non-inflammatory processes. Rather, astrocytes seem to play an important role on a cellular level, particularly in the case of ALS and PD. Interestingly, ARE regulated Nrf2 dependent genes are also preferentially activated in astrocytes thus providing an interesting cellular and molecular link between astrocyte dysfunction and the role of oxidative stress in neurodegeneration.

### 3.1. Alzheimer’s Disease

Alzheimer’s disease (AD) is a complex and genetically heterogeneous disease, characterized by cognitive impairment, memory deficits and later also personality changes. These alterations are associated with structural abnormalities, which in advanced stages are displayed as atrophy in MRI and CT scans of the brain. Loss of neurons in the neocortex, hippocampus and other subcortical regions of the brain are common features of AD [26]. Mitochondrial dysfunction and increased ROS and RNS production by reactive glial cells are considered to promote oxidative damage in lipids, DNA and proteins [27–31]. In tissue probes of AD patients comprising the temporal cortex and hippocampus, HO-1 expression is significantly elevated as compared to non-demented patients [28,32]. Moreover, expression of NAD(P)H dehydrogenase [quinone] 1 (NQO1) is increased in neurons and astrocytes in AD patients [33,34]. NQO-1 is a member of the NAD(P)H dehydrogenase (quinone) family encoding a cytoplasmic 2-electron reductase which reduces quinones to hydroquinones. The enzymatic activity of this Nrf2 target gene prevents the reduction of quinones that results in the production of radical species and thus exerts anti-oxidative effects. Consistently, a positive Nrf2 immunostaining was detected in the cytoplasm of hippocampal neurons from individuals with AD [35]. In a mouse model of AD, decreased expression of mRNAs encoding Nrf2, NQO1, glutamate-cysteine ligase (GCL), the catalytic subunit (GCLC) and a modifier subunit (GCLM) correlates with increased accumulation of β-amyloid deposits [36] *In vitro*, Nrf2 activation protects neuronal cells against amyloid α peptide induced neurotoxicity, suggesting that activation of the Nrf2-ARE pathway might be a possible target for future therapies. Within the next months an investigator initiated therapeutic trial with fumarates in ALS will start recruitment.

### 3.2. Amyotrophic Lateral Sclerosis

ALS is the most common adult onset motor neuron disease involving degeneration of the first and second motor neurons in the motor cortex and the spinal cord [37]. The sporadic as well as hereditary forms share the same clinical motor features including progressive muscle weakness, atrophy and spasticity. There is no cure for this devastating disease and ALS inevitably leads to death within maximal 3–5 years due to a denervation of the respiratory muscles in the late stages of the disease. 10%–20% of the familial ALS forms are caused by a toxic gain of function mutation in the Cu/Zn-superoxide dismutase (SOD1) [38]. A rodent model with over-expression of mutated SOD1 shares many features with ALS [39,40]. Oxidative stress, glutamate excitotoxicity, defective axonal transport and mitochondrial dysfunction are all involved in the postulated toxic effect of mutated SOD1 [41–44]. While the exact mechanism underlying cell death of motor neurons remains unclear, the toxic environment requires the expression of mutated SOD1 not only in neurons but also in non-neuronal cells. Thus, expression of mutated SOD1 in glial cells significantly contributes to disease progression. In contrast, SOD1 expression in neuronal cells is rather associated with the onset of symptoms [45–47]. NO and superoxide are produced by motor neurons in response to apoptotic signals mediated via Fas and p75^NTR^ pathways [48–50]. Additionally, a strong glial reaction typically surrounds degenerating neurons and oxidative markers are present in these cells as well as in motor neurons [51]. These toxic effects are paralleled by decreased mRNA expression of Nrf2 in the motor cortex and spinal cord from ALS postmortem tissue samples [49]. In contrast, in the G93A SOD mouse model of ALS, no altered expression of Nrf2 and ARE-driven genes is found [52]. Despite these apparent discrepancies, it is clear that there is an increase of ARE driven gene transcripts in the spinal cord in this model upon disease onset: At this time point, an increase of HO-1 is detected which might protect neuronal degeneration in early stages of the disease, but will be overwhelmed by the impact of other mechanisms that drive apoptosis [53]. Transgenic mice expressing mutant SOD1 exclusively in astrocytes display an increased apoptosis rate, but fail to develop a phenotype [54]. Yet, astrocytes isolated from such animals expressing mutant SOD1 are toxic to co-cultured wild type neurons. Over-expression of Nrf2 completely reverses the toxicity of such astrocytes in motor neuron culture. Well in line with this observation, over-expression of Nrf2 under a GFAP promoter delays the onset of the disease onset and prolongs the lifespan in ALS mouse models [55]. In summary, Nrf2 associated elevated ARE genes expression in astrocytes may not directly influence the toxic effect of mutated SOD in astrocytes but instead may improve the ability of motor neurons to cope with toxicity via a paracrine mechanism. Here, increased glutathione secretion from astrocytes may play a role and may also lead to decreased microglial responses [55]. These data from ALS models contribute to the first *in vivo* evidence that Nrf2 activation may result in improved neuroprotection. Thus, Nrf2 activation still may constitute a suitable therapeutic target in ALS.

### 3.3. Parkinson’s Disease

Parkinson’s disease (PD) is associated with neuronal loss of dopaminergic neurons in the substantia nigra pars compacta and in other brainstem regions which are involved in disease pathogenesis. Decreased mitochondrial activity and increased lipid- and DNA oxidation are detected in individuals with PD. Studies of familial PD have emphasized the relevance of mitochondrial dysfunction in the pathogenesis of the disease [56–58]. These observations are confirmed by studies in Nrf2 knock-out mice which display a greater loss of dopaminergic afferents in the striatum. Conversely, the implantation of Nrf2 over-expressing astrocytes protects against 6-hydroxydopamine in the mice [59,60]. Normally, Nrf2 deficiency results in increased 1-methyl-4-phenyl-1,2,3,6-tetrahydroxydopamine (MPTP) sensibility. Furthermore Nrf2 over-expression in astrocytes of mice on an otherwise Nrf2 knockout background completely rescues the toxic effect of MPTP [61]. Thus Nrf2 activation strengthens the anti-oxidant potential and protects against the neurotoxic dopamine analogues 6-hydroxydopamine and MPTP *in vivo* [60,62,63]. In line with this concept, the Nrf2 dependent factors NQO1 and HO-1 are strongly up-regulated in glial cells in postmortem tissue from PD patients, possibly indicating a naturally occurring, but insufficient anti-oxidative response as part of an endogenous rescue strategy in the CNS [64,65].

### 3.4. Huntington’s Disease

Huntington’s disease (HD) is another inherited progressive neurodegenerative disease. In HD, the molecular pathophysiology finally resulting in neurodegeneration is only partially understood and there is no established protective treatment so far. Some lines of evidence speak for the contribution of oxidative stress to neuronal damage, but the exact mechanism is still unknown. In the striatum of post-mortem HD brains, mitochondrial complex II, III, and IV activity are reduced [66,67]. In simple experimental models of mitochondrial complex II toxicity, where ROS production is augmented upon disruption of the electron transport, Nrf2 activation is protective. Nrf2 knockout mice are more sensitive to striatal lesions caused by administration of a complex II inhibitor [68,69]. In such a lesion paradigm, activation or over-expression of Nrf2 reduces lesion size [70]. Regarding cell types involved, especially astrocytes may exert neuroprotective effects: Injection of Nrf2 over-expressing astrocytes into the striatum results in protective effects against malonate toxicity *in vivo* [68]. Mitochondrial dysfunction and oxidative stress also play an important role in genetic models of HD. In the R6/2 mouse model of HD, a reduction of complex IV activity as well as an up-regulation of NO and superoxide is observed [71,72]. In this paradigm, Nrf2 dependent protective pathways may play a role, as recently revealed in a therapeutic study with dimethylfumarate (DMF; see below, [73]).

In summary, modulation of the Nrf2 pathway may constitute a promising target for various neurodegenerative disorders. The expression of phase II detoxification enzymes is governed by a Nrf2 dependent regulating element known as anti-oxidant response element (ARE). Over-expression of Nrf2 can thereby increase the endogenous anti-oxidant capacity of the brain thus rendering protection against oxidative stress in neurodegenerative disorders.

## 4. Oxidative Stress and the Nrf2 Pathways in Multiple Sclerosis

**Figure 2:**
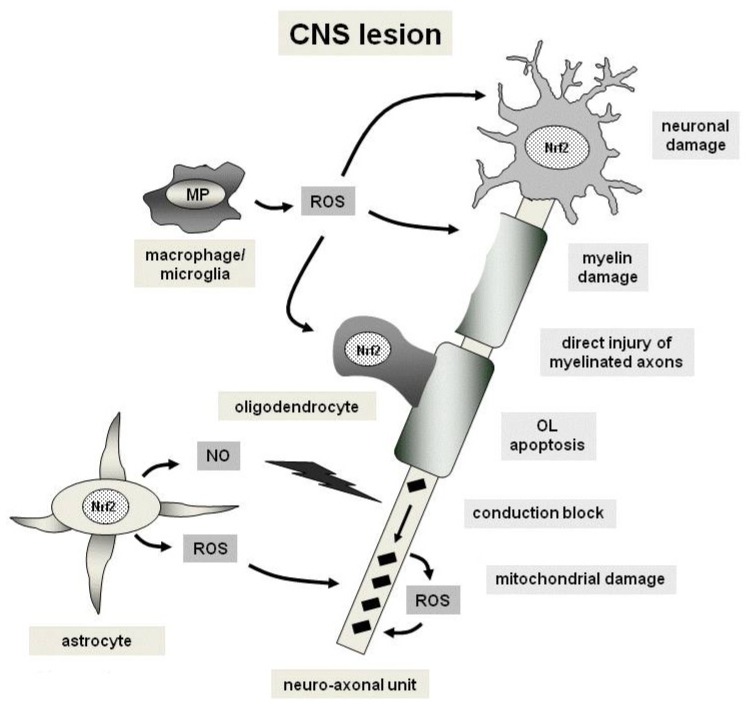
Mechanisms of oxidative injury and cytoprotection in a demyelinating Central Nervous System (CNS) lesion. Free radicals comprise nitric oxide (NO) and reactive oxygen as well as nitrogen species (Reactive Oxygen Species (ROS) or Reactive Nitrogen Species (RNS), respectively) which are mainly produced by macrophages, microglia and astrocytes. ROS and RNS lead to damage of neurons, axons, myelin and oliogdendrocytes (indicated by arrows). This process also may involve mitochondrial damage. Black squares indicate mitochondria which accumulate in injured axons. The cytoptrotective transcription factor Nrf2 is present in neurons, oligodendrocytes and astrocytes as part of the cellular anti-oxidative response. Abbreviations: OL, oligodendrocyte; MP, myeloperoxidase.

Multiple sclerosis (MS) is a chronic inflammatory disease of the CNS affecting both white and grey matter. Histopathologically, MS is characterized by focal demyelinating lesions throughout the white matter, which frequently coincide with cortical demyelination and which may be preceded by apoptotic destruction of oligodendrocytes [74,75]. Additionally, axonal damage is a key feature of MS pathogenesis that best correlates with permanent neurological deficits in patients. In general, axonal damage is observed in early as well as chronically demyelinating lesions and, in acute stages, is associated with the number of infiltrating immune cells. Inflammatory processes with infiltrating leukocytes play a crucial role in the pathology of the MS lesion mediated by the production of inflammatory mediators which also involve reactive oxygen species. Yet, also non-inflammatory mechanisms such as mitochondrial dysfunction may contribute to neurodegeneration in MS [76]. Excessive release of free radicals may play an important role in MS pathogenesis and promote transendothelial leukocyte migration as well as contribute to oligodendrocyte damage and as axonal degeneration [77–80]. Thus, oxidative stress stemming from different cell types and targeting several cellular components of the CNS to a variable extent is involved in this detrimental concert (Figure 2).

### 4.1. Mitochondrial Dysfunction

Interestingly, tissue alterations present in MS patients are strikingly similar to those presented in acute lesions of patients with white matter stroke [81]. These data suggest that energy deficiency may play an important role in the pathophysiology of neurodegenerative processes in MS patients. In line with this concept, mitochondrial injury with subsequent energy failure is declared as one of the main non-immune cell derived mechanisms which contributes to MS pathogenesis [82–84]. Mitochondrial disturbance is found in MS lesions by microarray based gene expression analysis and also with histological approaches [76,85–87]. With the help of MR spectroscopy, imaging studies may also provide new insights in energy metabolism in MS *in vivo*. Analysis of N acetyl aspartate, phosphorous and sodium allows for a direct assessment of energy availability and might offer new information on MS pathophysiology [88].

In active lesions, first mitochondrial changes are characterized by a dominant loss of cytochrome C oxidase 1 (COX1) and loss of complex IV activity in the mitochondrial respiratory chain [87]. In chronic inactive lesions, expression of mitochondrial numbers and activity is increased, possibly indicating the higher energy demand of demyelinated axons as compared to myelinated axons [89]. In MS patients, changes in circulating metabolites of energy metabolism correlates with disease progression [90]. Furthermore, increased extra-mitochondrial glucose metabolism as well as a depletion of ATP metabolites are found in the CSF of MS patients. These findings implicate mitochondrial dysfunction in the pathogenesis of disease progression and suggest a high energy demand in MS [91,92].

Thus, mitochondrial dysfunction associated tissue damage can be induced by at least three different mechanisms:

1. Energy failure
2. Induction of apoptosis: mitochondrial injury can elicit pro-apoptotic events via liberation of apoptosis induced factor or cytochrome C [93], and
3. Enhanced production of reactive oxygen species.

On the other hand, mitochondria themselves are highly susceptible for oxidative stress. Direct affection of the respiratory chain or induction of pro-apoptotic mechanisms in mitochondria can activate a cascade which finally leads to mitochondrial malfunction. NO and ROS mainly target respiratory chain complexes at different levels thereby inducing mitochondria transition and consecutively triggering apoptotic cascades [78,94]. In summary, these data at least in part explain the profound mitochondrial damage in MS lesions.

### 4.2. Free Radicals

Several studies have analyzed free radical induced tissue injury in MS lesions via biochemical methods and by immunocytochemical identification of oxidized nucleotides, proteins and lipids [95]. There are several sources of ROS and RNS in MS lesions. Production of RNS is dependent upon functional expression of the NO synthases 1–3 in different cell types [96]. ROS are mainly derived from activated macrophages and microglia [97]. Additionally, mitochondrial injury by itself enhances ROS production and oxidative injury (see above and [98]). The source of NO species (NOS) expression in MS lesions are again activated microglia and macrophages, mainly at the edge of active lesions [99,100]. Furthermore, myeloperoxidase activity in macrophages and microglia is found in actively demyelinating white matter and cortical lesions [101–103]. Members of the NADPH oxidase (NOX) family, particularly NOX2, are mainly responsible for the oxidative burst of macrophages and microglia. These data suggest that NOX activation and subsequent ROS formation represents an important pathomechanism of macrophage- and microglia-mediated neuronal and oligodendrocyte injury in MS [104].

In an elegant imaging model of EAE, intra-axonal mitochondrial defects, which already occur in the absence of demyelination, are shown to be an essential step towards axonal degeneration and local accumulation of invading macrophages. Here, exogenous anti-oxidants are able to reduce mitochondrial pathology and thereby mitigate axonal degeneration [105]. In chronic lesions, increased complex IV densities indicate a compensatory mechanism of action for the observed dysfunction of the respiratory chain. Likewise, increased mitochondrial density and complex IV activity are observed in remyelinating axons [106].

## 5. Anti-Oxidative Effects of Fumaric Acid Esters in Autoimmune Demyelination

In view of the pivotal role of oxidative stress in MS disease pathogenesis and the well described protective effects of Nrf2 pathway activation, pharmacological modulation of Nrf2 is an interesting therapeutic target in MS. Indeed, many compounds may experimentally induce Nrf2 activity *in vitro* and possibly also in animal models [61,72,73,107–125]. Yet, the safety profile of many of these compounds is unclear and application in clinical practice cannot be advised to date. This situation is different with fumaric acid esters (FAE) which constitute a well-established therapy for psoriasis in German speaking countries with a comprehensive safety profile of over 160,000 patient years. In the EAE model, application of FAE leads to neuroprotective effects which are mediated via Nrf2 and involve direct modification of Keap1 [126]. *In vitro*, FAE increase murine neuronal survival and protect human or rodent astrocytes against oxidative stress. In the chronic phase of EAE, preventive or therapeutic application of FAE ameliorates the disease course and improves preservation of myelin, axons and neurons. Treatment with FAE leads to stabilization of Nrf2 and activation of Nrf2 dependent transcriptional activity and expression of NQO-1 as target gene (see above). Moreover, the immediate metabolite of the most important FAE dimethylfumarate (DMF), monomethylfumarate (MMF), leads to direct modification of the inhibitor of the Nrf2, Keap-1. Activation of the Nrf2 pathway and the evidence that Nrf2 function is necessary for the effect of FAE suggest that the neuroprotective effects of FAE involve activation of Nrf2-mediated oxidative stress response mechanisms previously implicated as important for protection of the CNS in a variety of pathological conditions. Here, the ability of DMF to activate Nrf2 underpins the cytoprotective modality that further augments the natural anti-oxidant responses in MS tissues which is not yet targeted by other MS therapies [126].

Yet, it is still not clear which specific antioxidant pathway is mainly responsible for the protective effects in MS patients. There are even *in vitro* data, which reveal reduced glutathione levels in FAE treated astrocytes thus reflecting the complexity of oxidative stress pathways in CNS cells [127].

A very recently published study further characterizes the potential neuroprotective as well as cytoprotective effects of DMF and MMF on cellular resistance to oxidative damage in primary culture of CNS cells. DMF and MMF treatment increase the cellular redox potential, glutathione as well as ATP levels, and the mitochondrial membrane potential in a concentration dependent manner. Similarly, DMF as well MMF improve cell viability after ROS challenge. This effect is lost in cells that have eliminated or reduced Nrf2 levels. These data suggest that DMF and MMF are cytoprotective for neurons and astrocytes against oxidative stress induced cellular injury and loss via up-regulation of a Nrf2 dependent anti-oxidant response. This effect might be mediated via an improved mitochondrial function [128].

In a dose dependent manner, DMF also promotes protection of primary cortical neurons as well as hippocampal HT22 cells from glutamate toxicity [129]. This effect reaches its maximum after 24 h of incubation. In contrast MMF protection takes longer and becomes evident after 96 h of incubation. DMF increases Nrf2 protein abundance below dosages of 10 μM. Interestingly, DMF exerts protection even tough glutathione synthesis is blocked, suggesting that it enhances glutathione recycling. Neuroprotective concentrations of DMF also suppress levels of pro-inflammatory cytokines from activated splenocytes without exerting any effects on cell viability. The induction of an anti-oxidant response leading to glutathione synthesis seems to be the consequence of an initial and short-termed electrophilic response, since DMF decreased the glutathione content immediately after its addition to the cells. Nrf2 also contributes to the long-term effect of DMF in neuronal cells which may also involve other reported mechanisms such as the inhibition of the nuclear translocation of NF-κB. Besides the anti-oxidant effects, additional direct effects of DMF on immune cells were observed. These data involve effects on cytokine production of activate murine splenocytes and also (possibly secondary) effects on macrophage infiltration in EAE [119,129]. Together these data suggest an additional effect of DMF on immune cells which is different from its effect during the priming of a T cell response.

As previously mentioned, neurodegenerative diseases like HD are characterized by pathomechanisms also encompassing oxidative stress. In two mouse models of HD, DMF exerts cytoprotective effects and beneficial clinical effects on survival time and motor function. In the R6/2 model of HD, the clinical efficacy is corroborated by preservation of intact neurons and less pronounced dark cell degeneration in the striatum and motor cortex as well as an up-regulation of the transcription factor Nrf2 in striatal neurons [71].

These beneficial pharmacodynamic effects of FAE translate into a promising clinical trial situation for FAE in relapsing remitting MS. BG00012 is an oral formulation of DMF with improved gastroenteric coating and better side effect profile, which has already shown its beneficial effects for the treatment of relapsing remitting MS in multicenter phase II and III trials. In a phase II trial, treatment with BG00012 resulted in a reduced relapse rate in MS patients and positively influenced magnetic resonance marker indicative of inflammation as well as axonal destruction [109,110]. At present, promising data from two recently finished phase III trials have been presented at several international meetings and will soon appear in print form. Well in line with comprehensive cytoprotective effects of FAE, protective effects of FAE via the Nrf2 pathway were recently also published for the cardiovascular system [130].

## 6. Discussion

Oxidative stress is one of the hallmarks of tissue damage and plays an important role in neurodegenerative disease. In inflammatory diseases such as MS, oxidative stress mainly affects the degenerative phase of the disease which dominates later stages of the disease course. Oxidative damage also provides a good explanation for non-inflammatory aspects of MS pathogenesis. Such a mechanism may explain the preferential destruction of small calibre axons and the demise predominantly in axons with low mitochondrial content and larger axonal surface area [33]. In summary, highly reactive free radicals seem to be involved in inducing and amplifying tissue injury in the initial stage of MS lesion formation but also during progressive expansion or in diffuse injury of the normal appearing white matter. BG00012 is a promising novel oral therapeutic option in patients with relapsing-remitting MS shown to reduce disease activity and progression. These effects originate from an innovative combination of immunomodulatory and neuroprotective mechanisms.

## 7. Conclusions

Oxidative stress is thought to promote tissue damage in MS. FAE enhance cellular resistance to free radicals via Nrf2, a transcriptional factor and could prove useful for MS treatment. In this context, Nrf2 plays an important role of anti-oxidative pathways for tissue protection. Furthermore, FAE are a new, orally available treatment option which targets the NRf2 pathway and which have already shown their efficacy in phase II/III MS trials.
